# Vonoprazan-based bismuth quadruple therapy for first-line *Helicobacter pylori* eradication: A real-world, retrospective study

**DOI:** 10.1097/MD.0000000000046743

**Published:** 2026-01-02

**Authors:** Jin-Yan Zhang, Yan-Qing Wang, Wei-Feng Huang, Xiao-Yi Lei, Ji Li, Li-Yun Guo, Xiao-Fen Zhang

**Affiliations:** a Department of Gastroenterology and Hepatology, The First Affiliated Hospital of Xiamen University, School of Medicine, Xiamen University, Xiamen, China; b The School of Clinical Medicine, Fujian Medical University, Fuzhou, Fujian Province, China; c Department of Ultrasound, The First Affiliated Hospital of Xiamen University, School of Medicine, Xiamen University, Xiamen, China.

**Keywords:** first-line therapy, *Helicobacter pylori*, quadruple therapy, vonoprazan

## Abstract

The rising prevalence of drug-resistant *Helicobacter pylori (H. pylori*) strains necessitates the development of more effective treatment approaches for eradication, such as bismuth quadruple therapy (BQT). We aimed to evaluate the efficacy and safety of vonoprazan (VPZ)-based BQT as a first-line regimen for *H. pylori* eradication. We conducted a retrospective analysis of consecutive treatment-naïve patients with *H. pylori* infection who received VPZ-based BQT between March 2022 and September 2023. Patients received a 14-day BQT regimen consisting of VPZ (20 mg), bismuth potassium citrate (240 mg), amoxicillin (1.0 g), and clarithromycin (500 mg) twice daily. Demographics, eradication rates, compliance, and adverse events (AEs) were assessed. 231 patients were included in the study, with 87 males (37.7%) and an average age of 40.84 ± 10.72 years old. The eradication rates for the intention-to-treat, modified intention-to-treat, and per-protocol analyses were 92.2% (213/231; 95% confidence interval [CI] 88.7–95.7%), 97.3% (213/219; 95% CI 95.1–99.4%), and 99.1% (210/212; 95% CI 97.7–100.0%), respectively. Patient compliance with the treatment was 97.0% (224/231). The incidence of AEs was 22.1% (51/231). Most AEs were mild and tolerable. Poor compliance was found to be an independent factor predicting treatment failure. This study demonstrated that the 14-day VPZ-based BQT as a first-line treatment regimen was highly effective and safe for eradicating *H. pylori* in a Chinese setting, with excellent patient compliance.

## 1. Introduction

*Helicobacter pylori (H. pylori*) infection is a significant global health issue and a major risk factor for various gastrointestinal diseases, including chronic atrophic gastritis, peptic ulcer diseases, gastric mucosa-associated lymphoid tissue lymphoma, and gastric adenocarcinoma, as well as some extragastric disorders such as idiopathic thrombocytopenic purpura and iron deficiency anemia.^[1,2]^ Successful eradication of *H. pylori* is essential in preventing and treating these diseases. Currently, the most widely used treatment regimen for *H. pylori* infection is proton-pump inhibitor (PPI)-based triple or quadruple therapy, consisting of a PPI plus 2 antibiotics with or without bismuth. However, the efficacy of these therapies is limited, with success rates below 80% in some regions, mainly due to antibiotic resistance.^[3,4]^ In addition, these regimens are usually associated with various adverse reactions such as nausea, diarrhea, abdominal pain, headache, and taste disturbance, which may affect patients’ compliance and quality of life. Therefore, it is necessary to search for more effective and safe treatment options for *H. pylori* infection.

Vonoprazan (VPZ) is a novel potassium-competitive acid blocker (P-CAB) that was approved in Japan for *H. pylori* eradication in February 2015.^[5]^ Compared to conventional PPI, VPZ provides longer-lasting and stronger suppression of gastric acid secretion, making it a potential candidate for improving the effectiveness of *H. pylori* eradication regimens.^[6]^ Several studies have investigated the efficacy of VPZ-containing dual or triple therapies for *H. pylori* infection, but the findings were inconsistent. Studies conducted in Japan have reported that VPZ dual therapy achieved high eradication rates of up to 93%, which were comparable to those of VPZ triple therapy.^[7–9]^ In contrast, a phase 3 clinical trial from the United States and Europe revealed that in the full analysis set, the eradication rates of VPZ triple and dual therapy were 80.8% and 77.2%, respectively.^[10]^ Another study performed in Lanzhou of China, also showed that VPZ-based dual and triple therapies did not achieve satisfactory results.^[11]^ Given these uncertain outcomes, we need to explore and implement alternative VPZ-based strategies.

Currently, there is little evidence of the clinical outcomes of VPZ-based bismuth quadruple therapy (BQT). Therefore, this study aimed to evaluate the efficacy and safety of VPZ-based BQT (VPZ, amoxicillin, clarithromycin, and bismuth) as a first-line regimen for *H. pylori* eradication, based on real-world data.

## 2. Methods

### 2.1. Study design

This was a single-center, 1-arm, and retrospective analysis of prospectively followed consecutive treatment-naïve patients with *H. pylori* infection who received VPZ-based BQT between March 2022 and September 2023 at the Department of Gastroenterology of The First Affiliated Hospital of Xiamen University. We collected information on patient demographics, upper endoscopic examination findings, adverse events (AEs), compliance, and treatment results. This study was approved by the institutional ethics committee (No. 2023-046) and conducted in accordance with ethical principles detailed in the Declaration of Helsinki. Written informed consent was waived due to the retrospective design of this study. All authors had access to the data and were involved in the review and approval of the final manuscript.

### 2.2. Study populations

Study participants were eligible for enrollment if they were between 18 to 70 years old, had a confirmed diagnosis of *H. pylori* infection through carbon-13/14 urea breath test (^13^C/^14^C-UBT), and were treatment-naïve. Patients meeting the following criteria were excluded: previous history of *H. pylori* eradication therapy; previous history of gastric surgery; severe comorbidities or mental illnesses; those who had taken H_2_ receptor antagonists, PPIs, or P-CABs within the previous 2 weeks, and those who had used bismuth and antibiotics within the preceding 4 weeks; pregnant or lactating women.

### 2.3. Treatment regimen

All patients received a 14-day VPZ-based BQT regimen consisting of VPZ (Tianjin Takeda Pharmaceutical Co., Ltd, Tianjin, China) 20 mg twice daily, bismuth potassium citrate (Hunan Warrant Chiral Pharmaceutical Co., Ltd, Changsha, China) 240 mg twice daily, amoxicillin (Zhejiang Jinhua CONBA Bio-pharm. Co., Ltd, Zhejiang, China) 1.0 g twice daily, and clarithromycin (HEC Pharmaceutical Co., Dongwan, China) 500 mg twice daily. VPZ and bismuth potassium citrate were taken 30 minutes before meals. Amoxicillin and clarithromycin were taken 30 minutes after meals.

### 2.4. Patient education

Before initiating therapy, all study participants received both oral and written instructions, covering the following topics: the clinical importance of *H. pylori* eradication; the significance of medication adherence; drug names, dosages, frequencies of administration, and treatment duration; possible AEs associated with the treatment; dietary and lifestyle guidance; and follow-up arrangements. Additionally, patients were invited to join a WeChat group for management purposes, and real-time consultation was available for any questions during medication usage.

### 2.5. Study outcomes

The primary endpoint of this study was the *H. pylori* eradication rates. The efficacy of treatment was assessed by performing ^13^C-UBT at 6 to 8 weeks after completion of therapy. Participants were instructed to avoid taking any H_2_ receptor antagonists, PPIs, or P-CABs 2 weeks before the ^13^C-UBT and any antibiotics 4 weeks before the test. A negative ¹³C-UBT result (<4‰) indicated successful eradication of *H. pylori*. The secondary outcomes were patient compliance and the incidence of AEs. Compliance and AEs were evaluated using a standardized questionnaire administered at the end of treatment. Good compliance was defined as having taken >80% of the prescribed drugs. AEs were categorized into 3 groups based on the degree of interference with daily activities: mild (no interference with usual activities), moderate (some interference with usual activities), and severe (considerable interference with usual activities).

### 2.6. Statistical analysis

Eradication rates of *H. pylori* were assessed using intention-to-treat (ITT), modified intention-to-treat (mITT), and per-protocol (PP) analyses. The ITT analysis included all study participants who took at least 1 dose of medicine, irrespective of their adherence to protocol or discontinuation of treatment. In the mITT analysis, participants who had received at least 1 dose of medication and obtained ^13^C-UBT results posttreatment were included. The PP analysis involved patients who had taken >80% of the prescribed medication and completed follow-up testing. Eradication rates along with their corresponding 95% confidence interval (CI) were calculated. Continuous variables were presented as mean with standard deviation or median with interquartile range. Categorical variables were reported as numbers and percentages. The differences between the groups were analyzed using either the chi-square test or Fisher’s exact test for categorical variables, and either the Student *t* test or a nonparametric test for continuous variables. Univariate analysis was conducted to identify which variables had a significant predictive impact on *H. pylori* eradication. If this analysis revealed 2 or more variables with statistical significance, multiple logistic regression analysis was performed using a backward modeling strategy. A *P*-value of <.05 was considered statistically significant. SPSS software package (version 16; IBM, Armonk) was employed for data analyses.

## 3. Results

### 3.1. Baseline characteristics of patients

A total of 231 patients were included in the study, comprising 87 males (37.7%) and 144 females (62.3%). The average age was 40.84 ± 10.72 years, and the mean body mass index (BMI) was 22.26 ± 3.19 kg/m^2^. Among them, 19 (8.2%) and 49 (21.2%) patients had a history of smoking and alcohol consumption, respectively. Additionally, 107 (46.3%) patients habitually drank tea, and 59 (25.5%) drank coffee. None of the cases had a family history of gastric cancer. Most of the subjects (n = 159, 68.8%) received treatment for *H. pylori* infection discovered through a positive ^13^C-UBT or ^14^C-UBT during a routine health examination, while some patients underwent eradication therapy for other reasons: chronic gastritis (n = 39), gastric ulcer (n = 3), duodenal ulcer (n = 26), and complex ulcer (gastric and duodenal ulcer, n = 4). Patient demographics and baseline clinical characteristics are presented in Table 1.

**Table 1:** Patient demographics and baseline clinical characteristics.

Characteristics, no. (%)	Total patients (n = 231)
Age, mean ± SD, yr	40.84 ± 10.72
Gender, male	87 (37.7)
BMI, mean ± SD, kg/m^2^	22.26 ± 3.19
Cigarette smoking	19 (8.2)
Alcohol intake	49 (21.2)
Tea drinking	107 (46.3)
Ingestion of coffee	59 (25.5)
History	
Hypertension	14 (6.1)
Diabetes	6 (2.6)
Diagnosis	
Chronic gastritis	39 (16.9)
Gastric ulcer	3 (1.3)
Duodenal ulcer	26 (11.3)
Complex ulcer	4 (1.7)
Health examination	159 (68.8)

BMI = body mass index, SD = standard deviation.

### 3.2. Treatment efficacy and compliance

**Figure 1.**
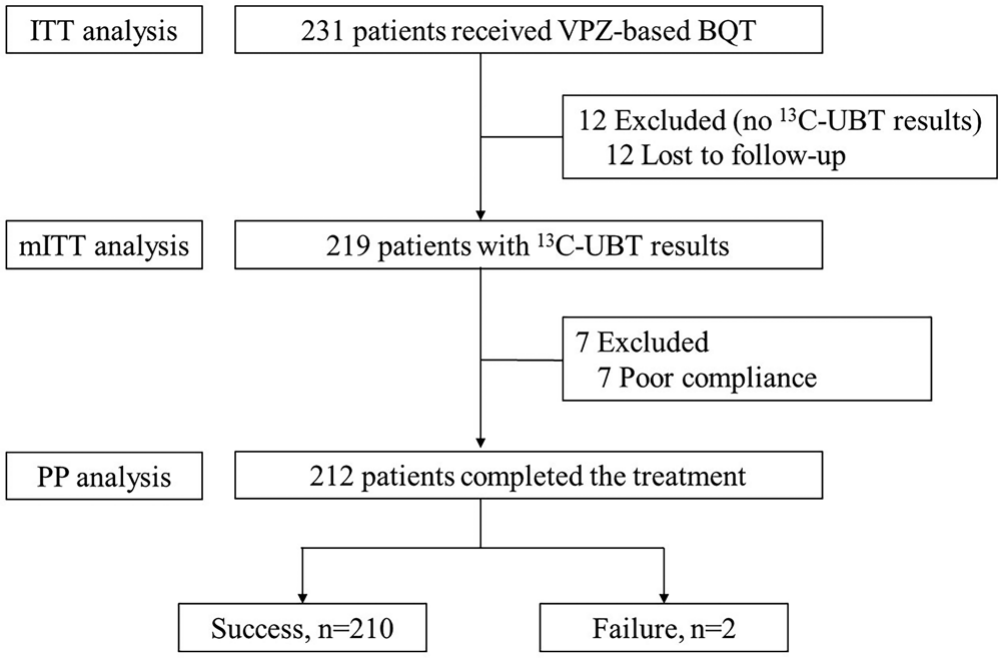
Flowchart of the study. BQT = bismuth quadruple therapy, ^13^C-UBT = carbon-13 urea breath test, ITT = intention-to-treat, mITT = modified intention-to-treat, PP = per-protocol, VPZ = vonoprazan.

**Figure 2.**
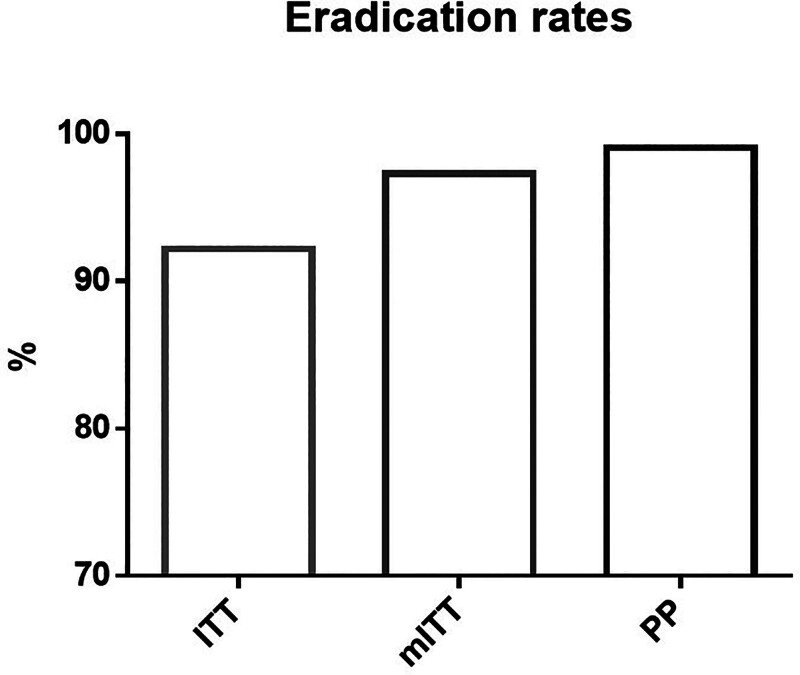
The eradication rates for the ITT, mITT, and PP analyses. ITT = intention-to-treat, mITT = modified intention-to-treat, PP = per-protocol.

Fig. 1 depicts the flowchart of this study. Out of the included subjects, 12 patients were lost to follow-up and thus excluded from the mITT analysis. Among the remaining participants, 7 were further excluded from the PP analysis due to poor compliance – 3 patients discontinued medication because of drug allergies after 8 to 10 days, and 4 patients frequently forgot to take their medication. Finally, a total of 212 patients were included in the PP analysis, where 210 of them achieved successful eradication. Among the 7 patients with poor compliance, 3 achieved successful eradication. Based on these findings, the eradication rates for the ITT, mITT, and PP analyses were 92.2% (213/231; 95% CI 88.7–95.7%), 97.3% (213/219; 95% CI 95.1–99.4%), and 99.1% (210/212; 95% CI 97.7–100.0%), respectively (Fig. 2). Good compliance was achieved in 97.0% (224/231) of patients.

### 3.3. AEs

The incidence of AEs in the study was 22.1% (51/231), including 48 cases of mild AEs and 3 of moderate AEs. No severe AEs were reported. The main AEs included oral bitterness, nausea, diarrhea, abdominal discomfort, abdominal pain, skin rash, dizziness, and easy hunger (Table 2). All symptoms except for skin rash resolved without any intervention after the end of treatment. Three patients with skin rash were successfully treated using oral or topical antiallergic drugs. None of the patients required hospitalization due to AEs.

**Table 2:** Adverse events of vonoprazan-based bismuth quadruple therapy.

Variables, no. (%)	Total patients (n = 231)
Total AEs	51 (22.1)
AE grade	
Mild	48 (20.8)
Moderate	3 (1.3)
Severe	0 (0)
AE variety	
Oral bitterness	46 (19.9)
Abdominal discomfort	5 (2.2)
Abdominal pain	7 (3.0)
Nausea	10 (4.3)
Diarrhea	7 (3.0)
Skin rash	5 (2.2)
Dizzy	6 (2.6)
Easy hunger	3 (1.3)
Discontinued due to AEs	3 (1.3)
Compliance	224 (97.0)

AE = adverse event.

### 3.4. Predictors of successful H. pylori eradication

Univariate analysis revealed patients with good compliance had significantly higher rates of *H. pylori* eradication than those with poor compliance (*P* < .05). Age, gender, BMI, cigarette smoking, alcohol intake, tea drinking, ingestion of coffee, history of hypertension or diabetes mellitus, and diagnosis did not have a significant effect on eradication rates (Table 3). The multivariable analysis showed that poor compliance was a risk factor for successful *H. pylori* eradication (Table 4).

**Table 3:** Variate analysis of the factors that influence the efficacy of *Helicobacter pylori* eradication.

Variables, no. (%)	Eradication success (n = 213)	Eradication failure (n = 6)	*P*-value
Age			.540
<35 years	71 (33.3)	3 (50.0)	
35–55 years	116 (54.5)	3 (50.0)	
>55 years	26 (12.2)	0 (0)	
Gender			.552
Male	79 (37.1)	5 (83.3)	
Female	134 (62.9)	1 (16.7)	
BMI			.444
<22.0 kg/m^2^	107 (50.2)	4 (66.7)	
22.0–25.0 kg/m^2^	61 (28.6)	2 (33.3)	
>25.0 kg/m^2^	45 (21.1)	0 (0.0)	
Cigarette smoking			1.000
Yes	17 (8.0)	0 (0.0)	
No	196 (92.0)	6 (100.0)	
Alcohol intake			.466
Yes	44 (20.7)	0 (0.0)	
No	169 (79.3)	6 (100.0)	
Tea drinking			.860
Yes	97 (45.5)	2 (33.3)	
No	116 (54.5)	4 (66.7)	
Ingestion of coffee			.974
Yes	55 (25.8)	1 (16.7)	
No	158 (74.2)	5 (83.3)	
Hypertension			1.000
Yes	11 (5.2)	0 (0)	
No	202 (94.8)	6 (100.0)	
Diabetes mellitus			1.000
Yes	5 (2.3)	0 (0)	
No	208 (97.7)	6 (100.0)	
Diagnosis			.741
Peptic ulcer	28 (13.1)	0 (0)	
Non-ulcer or health examination	185 (86.9)	6 (100.0)	
Compliance			.000
Good	210 (98.6)	2 (33.3)	
Poor	3 (1.4)	4 (66.7)	

BMI = body mass index.

**Table 4:** Multivariate analysis of the factors that influence the efficacy of *Helicobacter pylori* eradication.

Factors	Multivariate analysis	
	OR (95% CI)	*P*-value
Gender		
Female vs male	7.131 (0.192–264.483)	.297
Cigarette smoking		
Yes vs no	0.000	.999
Alcohol intake		
Yes vs no	0.000	.998
Tea drinking		
Yes vs no	0.873 (0.040–18.931)	.931
Ingestion of coffee		
Yes vs no	0.369 (0.014–9.821)	.552
Diagnosis		
Ulcer vs non-ulcer or health examination	0.000	.998
Compliance		
Good vs poor	210.034 (14.224–3101.457)	.000

CI = confidence interval, OR = odds ratio.

## 4. Discussion

We conducted a real-world, retrospective study of a VPZ-based BQT as first-line treatment for *H. pylori* infection. The results suggested that this 14-day treatment regimen comprising VPZ, amoxicillin, clarithromycin, and bismuth was effective and safe, with excellent patient compliance.

Although PPI-based BQT is currently recommended as a first-line *H. pylori* treatment regimen by various guidelines or consensus reports,^[12,13]^ its eradication efficacy has been declining in some regions. A multicenter clinical trial conducted in China showed that the eradication rates of 10-day PPI-based BQT (esomeprazole + bismuth + amoxicillin + clarithromycin) were 77.4% and 87.0% for the ITT and PP analyses, respectively.^[14]^ Another study performed in Iran revealed that 14-day PPI-based BQT (pantoprazole + bismuth + amoxicillin + clarithromycin) achieved eradication rates of 77.2% and 76.1% as determined by ITT and PP analyses, respectively.^[15]^ According to efficacy report cards proposed by Graham et al,^[16]^ the ITT eradication rates of these 2 regimens reached grade F (<80%), which were considered unacceptable.

There are various reasons for the failure of *H. pylori* eradication, including increased antibiotic resistance, poor patient compliance, and insufficient acid suppression. The susceptibility of *H. pylori* to antibiotics is affected by the intragastric pH level, which can alter the bioactivity and stability of antibiotics, as well as influence bacterial replication status.^[17,18]^ Some antibiotics, such as amoxicillin and clarithromycin, require *H. pylori* to be actively replicating for maximal antimicrobial effects. Thus, persistent control of intragastric pH plays a crucial role in improving *H. pylori* eradication rates. PPIs, the most commonly used acid inhibitors, exhibit a slow and cumulative acid suppression effect, necessitating multiple dosages to inhibit new proton-pump synthesis for maximal acid suppression.^[19]^ In addition, the metabolic and acid suppression properties of PPIs can be affected by genetic polymorphisms in the liver enzyme cytochrome P450 2C19 (CYP2C19).^[20]^ In recent years, a novel drug known as VPZ, which belongs to the class of P-CABs, has been developed. A number of studies have demonstrated that VPZ exerts more robust, rapid, and sustained inhibition of gastric acid secretion than PPIs,^[6,21]^ thereby offering a promising option for improving the efficacy of *H. pylori* eradication therapies.

Several studies have investigated the efficacy of VPZ-containing dual or triple therapies for *H. pylori* infection, but the findings were inconsistent. Meta-analyses of Asian trials have shown that VPZ-based triple therapy, consisting of VPZ, amoxicillin, and clarithromycin, achieved significantly higher eradication rates (ITT 91.4%; PP 92.6%) compared to PPI-based triple therapy (ITT 74.8%; PP 76.4%),^[22]^ including in patients infected with clarithromycin-resistant strains of *H. pylori*.^[23]^ A multicenter clinical trial conducted in China showed that the eradication rate of *H. pylori* in the VPZ-based dual therapy was 88.7% and 95.6% according to the ITT analysis and PP analysis, respectively.^[24]^ Another study in China also reported satisfactory eradication rates (>90%) with a 10-day VPZ-based dual therapy (VPZ 20 mg twice daily + amoxicillin 750 mg 4 times daily).^[25]^ However, a randomized clinical trial performed in the United States and Europe indicated that VPZ dual therapy and VPZ triple therapy had lower eradication rates of 77.2% and 80.8% (ITT analysis), and 81.1% and 85.7% (PP analysis), respectively.^[10]^ Possible factors contributing to differences in the eradication outcomes between Eastern and Western populations include race/ethnicity, variations in BMI, different treatment compliance, and genetic heterogeneity, warranting further investigation.

Currently, clinical studies on the application of VPZ-based BQT for *H. pylori* eradication are relatively scarce. A study with only 12 patients indicated that VPZ-based BQT achieved a 100% eradication rate.^[26]^ There are only 2 prospective studies on VPZ-based BQT. One study reported a successful eradication rate of 91.5% for *H. pylori.*^[27]^ In another study, a VPZ-based BQT regimen consisting of vonoprazan 20 mg daily with amoxicillin 1000 mg, furazolidone 100 mg, and colloidal bismuth 200 mg each given twice a day demonstrated eradication rates of 96.2% and 94.9% in the ITT analysis, and 98.6% and 97.4% in the PP analysis for treatment durations of 10 days and 14 days, respectively.^[28]^ In this study, we investigated the efficacy of 14-day VPZ-based BQT in 231 patients and found that the eradication rates were 92.2% in the ITT analysis, 97.3% in the mITT analysis, and 99.1% in the PP analysis. The results exceeded the clinically significant threshold of 90% for assessing the effectiveness of *H. pylori* treatment regimens. Achieving success rates above this limit can reduce the need for further therapy and decrease the likelihood of secondary antibiotic resistance.

In this study, we found that patients with good compliance had significantly higher rates of *H. pylori* eradication than those with poor compliance. Patient compliance is an important factor in determining treatment efficacy. Some patients may cease taking medication due to various reasons, including lack of motivation to continue treatment, forgetfulness, loss of drugs, worsening or relief of symptoms, and the occurrence of adverse reactions. According to a recent meta-analysis, the implementation of enhanced patient education through various communication strategies, such as telephone, short message service, and mobile apps, has shown promising results in improving adherence and ultimately leading to higher eradication rates.^[29]^ In this study, conventional instructions were provided orally and in writing for all patients before the initiation of therapy. In addition, we utilized the WeChat app to conduct enhanced patient education, promptly answer patient questions during treatment, and encourage patients experiencing mild adverse reactions to continue treatment. Excellent compliance was achieved in 97.0% (224/231) of patients, highlighting the significance of detailed patient education and effective patient management for good clinical outcomes.

Our study demonstrated that 14-day VPZ-based BQT was well-tolerated and safe. The AEs associated with *H. pylori* eradication therapy in prior studies mainly include oral bitterness, diarrhea, nausea, rash, and abdominal pain,^[30]^ which are consistent with our study findings. Of the 231 patients, 51 (22.1%) experienced AEs, most of which were mild in severity. No serious AEs were reported. The most frequently reported AE was oral bitterness, followed by nausea and abdominal pain. These symptoms do not require further intervention and disappeared spontaneously after completion of the treatment. In this study, 3 patients had to discontinue treatment on days 8 to 10 due to skin rash, but they quickly recovered after receiving anti-allergy treatment.

The main strength of this study is the largest sample size currently available for the evaluation of 14-day VPZ-based BQT. However, there are several limitations that need to be acknowledged. Firstly, although our data was extracted from a prospectively collected case database, this was a retrospective study, and potential selection or information biases might have been present. Secondly, the study was conducted in a single center, which may limit the generalizability of the findings to other settings. Further multicenter and prospective studies are warranted to confirm the external validity of our results. Thirdly, there was no control group for comparison, making it challenging to determine which component in the treatment regimen most affected eradication success. Finally, we did not investigate antibiotic resistance and its potential impact on *H. pylori* treatment. Resistance profiling is essential for determining whether treatment failure is attributable to the therapeutic regimen itself or to antimicrobial resistance. In China, resistance rates to clarithromycin, metronidazole, and levofloxacin are approximately 34%, 75%, and 35%, respectively.^[31]^ The absence of resistance data may overestimate the value of “ineffective regimens” that perform well in low-resistance populations but fail in high-resistance regions.

## 5. Conclusion

This study demonstrated that 14-day VPZ-based BQT as a first-line treatment regimen in a Chinese setting was highly effective and safe in the eradication of *H. pylori*, with excellent patient compliance. Further prospective, well-designed, randomized, and large-scale studies are needed to validate our findings.

## Acknowledgments

Thanks to Dr Hua Li, who has helped in our research and writing.

## Author contributions

**Writing – review & editing:** Jin-Yan Zhang, Yan-Qing Wang.

**Data curation:** Wei-Feng Huang, Li-Yun Guo.

**Validation:** Xiao-Yi Lei.

**Writing – original draft:** Ji Li.

**Supervision:** Xiao-Fen Zhang.
